# A Case of Idiopathic Colonic Intramural Hematoma: Successful Expectant Management

**DOI:** 10.7759/cureus.51330

**Published:** 2023-12-30

**Authors:** Taha Al-Mwald, Abdullah Mayas, Saif A Ghabisha, Saleh Al-wageeh, Faisal Ahmed

**Affiliations:** 1 Radiology, Ibb University, Ibb, YEM; 2 General Surgery, Ibb University, Ibb, YEM; 3 Urology, Ibb University, Ibb, YEM

**Keywords:** intramural hematoma, intestinal obstruction, case report, conservative treatment, intramural colonic hematoma

## Abstract

Intramural gastrointestinal hematomas are commonly observed following abdominal trauma or are associated with coagulopathy disorders. In contrast, idiopathic gastrointestinal hematoma is rare, and colonic involvement is sporadic, with very few published reports. We report the case of a 29-year-old female who presented with right hypogastric pain over the last three days. Abdominal CT with contrast revealed an 8.5 × 6 × 7.5 cm pre-occlusive intramural hematoma of the ascending colon up to the hepatic flexure with diffuse edematous wall thickening, indicating colonic obstruction. On colonoscopy, the site of the intramural hematoma was identified without active bleeding or obvious pathology, and the colonoscope successfully passed through the region. The patient was managed conservatively. A month later, abdominal CT revealed complete resolution of the colonic hematoma. After two months of follow-up, the patient was free from gastrointestinal symptoms. In conclusion, idiopathic colon intramural hematoma is rare, with a challenge in diagnosis and treatment; efforts should be made to treat it with conservative therapy.

## Introduction

Intramural gastrointestinal hematomas are rare; colonic involvement is sporadic, with very few published reports [1,2]. The explanation for this rarity is thought to be the protective function of taenia coli, which can prevent blood from diffusing into the intestinal wall if intramural arteries burst owing to physical trauma [3]. Radiological imaging, particularly CT scans with intravenous and oral contrast administration, may be used to diagnose accurately [3]. In the past, most patients with colonic intramural hematoma were surgically treated (laparoscopy or open procedures) [4]. However, conservative management, on the other hand, may be effective in the absence of serious circumstances, such as active bleeding, perforation, infection, bowel necrosis, gangrene, and so on [5]. Intramural hematomas can induce partial or full bowel blockage, and surgical therapy should be considered in patients who have intestinal necrosis, peritonitis, or a deteriorating overall status despite conservative treatments. Early diagnosis and treatment of hematoma can help to avoid intestinal blockage and surgery [3]. Herein, we present an idiopathic colonic intramural hematoma case in a 29-year-old female patient with an atypical clinical presentation.

## Case presentation

Patient information

A 29-year-old female presented with a three-day history of colicky abdominal pain, primarily in the right hypochondrium. The pain was associated with bilious vomiting and loose, bloody stools. There was no history of a fever or loss of appetite. The patient was not a smoker and denied any recent trauma, with no significant findings on her medical, surgical, or family history.

Clinical findings

During the presentation in our outpatient radiologic clinic, the patient was conscious and displayed normal vital signs (temperature: 37.5°C; respiratory rate: 14 breaths/min; blood pressure: 120/70 mmHg; pulse rate: 62 beats/min). Abdominal examination revealed abdominal distention with mild tenderness and guarding on palpation in the right upper quadrant. A digital rectal examination revealed an empty rectum with no detectable pathology.

Diagnostic assessment

**Figure 1:**
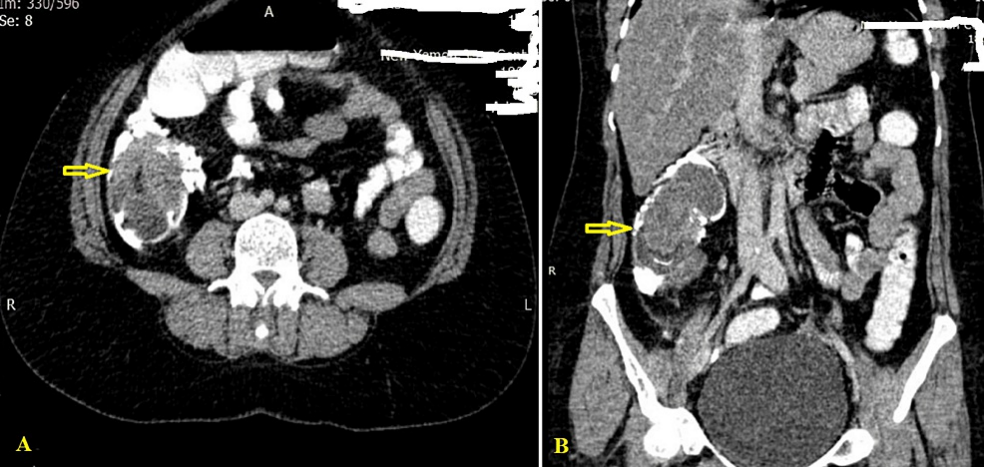
Abdominal/pelvic CT scan with intravenous, oral, and rectal contrast showing the intramural hematoma circumferential wall thickening with luminal stenosis at the ascending colon (arrow). (A) Axial view and (B) coronal view

The initial laboratory results showed a substantial WBC of 14.34 109/L, with 93.7% neutrophils and hemoglobulin of 9.3 gm/dL. The level of CRP was 8.21 mg/L (ref. 0.5 mg/dL). The PT as well as the international normalized ratio fell within the scope of normal values. All other blood tests and urine analysis results were within the normal ranges. Plain abdominal radiography was done, and there was no sign of obstruction or perforation. An abdominal CT scan using oral and intravenous contrast and rectal enema revealed an 8.5 × 6.0 × 7.5 cm pre-occlusive intramural hematoma of the ascending colon up to the hepatic flexure with diffuse edematous wall thickening, indicating colonic obstruction (Figure 1).

**Figure 2:**
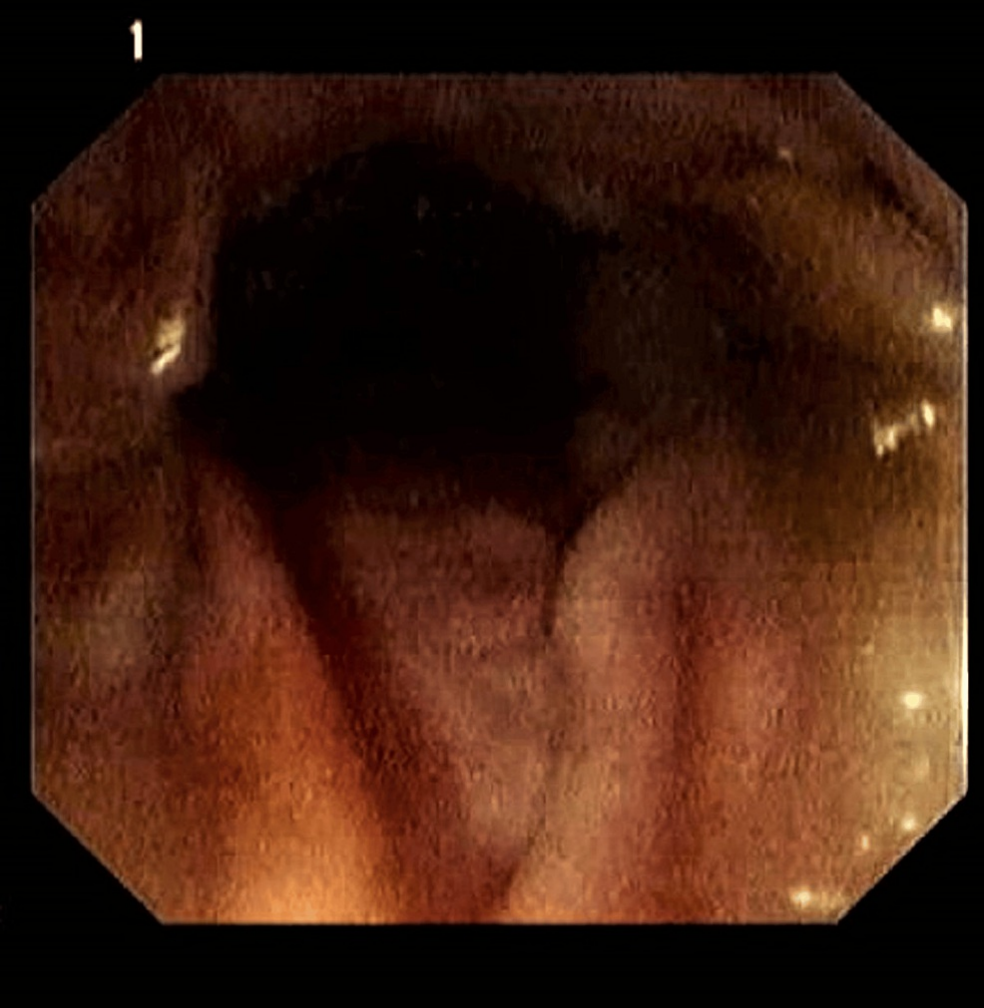
Colonoscopy images showing a normal appearance of the sigmoid section

On colonoscopy, the site of the intramural hematoma was identified without active bleeding or obvious pathology, and the colonoscope successfully passed through the region (Figure 2).

Therapeutic interventions

The patient was successfully treated with conservative management, including bowel rest (total parenteral nutrition), nasogastric tube drainage, intravenous fluid hydration, and antibiotic therapy (intravenous ciprofloxacin 400 mg every 12 hours and metronidazole 500 mg every eight hours) for three days.

Follow-up and outcome

**Figure 3:**
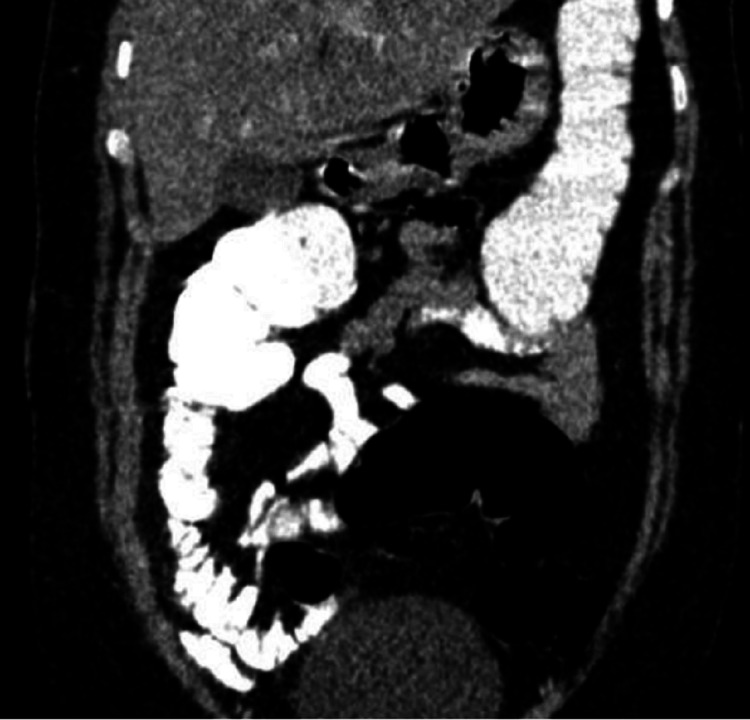
Abdominal/pelvic CT scan with contrast after one month indicating clear appearance without pathology

The patient’s symptoms completely resolved on the third day of admission. A follow-up abdominal CT scan one month later was normal, without any previous pathology (Figure 3).

After three months of follow-up, the patient was free of gastrointestinal symptoms.

## Discussion

Intramural hematoma may occur through the gastrointestinal tract, and the intestinal areas at the intersection of retroperitoneally fixed and non-fixed were the most typically impacted. The duodenum was the most typically implicated bowel area with traumatic acute intramural hematoma, followed by the ascending colon/caecum [3,4]. Isolated colon involvement is rare owing to the protective role of teniae coli, which usually inhibits the distribution of bleeding in the intestinal wall [1,6].

Blood reaching the intestinal wall from the mesentery as a consequence of crushing pressures causing shearing of bowel layers and rupture of terminal blood vessels is hypothesized to cause traumatic acute intramural hematoma. Another mechanism proposed for bowel intramural hematoma is fast decompression of splanchnic circulation due to reduced abdominal pressure, which causes the gut to rupture and bleed in situations of therapeutic anticoagulant medication [3,7]. The common etiologies of this condition are trauma and coagulopathy disorders [2]. Other uncommon etiologies are malignancies and chemotherapy [3,6]. Abdominal injuries and bleeding disorders such as hemophilia and leukemia are possible causes. They are highly rare as an iatrogenic injury such as double-balloon enteroscopy, stapled hemorrhoidopexy, polypectomy, or as a rare complication of vaginal birth [2,8]. Our patient has been recorded as an infrequent, sporadic, idiopathic case because no risk factors have been identified.

The main symptom is abdominal pain, which persists for several days before the emergence of symptoms of gastrointestinal obstruction [1]. Progression of symptoms and complications can occur from intramural hematomas, such as blood loss, bowel gangrene, intussusception, or obstruction, due to rupture of the hematoma in the acute phase, leading to hemodynamic instability [3]. However, symptoms of colonic intramural hematoma are less severe due to the large lumen, making intestinal obstruction more challenging [3].

As in our case, laboratory testing may reveal anemia and leukocytosis [3,9]. An elevated WBC count occurs due to hemorrhagic rupture of the bowel wall, which results in intramural and peritoneal distribution of gastrointestinal bacteria and subsequent infections. It should be noted that leukocytosis may be connected to a medical condition associated with sepsis. Furthermore, leukocytosis with a mass shown on a radiologic image may make hematoma identification more difficult because intraperitoneal contamination cannot be ruled out in such a scenario [6,9].

The diagnosis of gastrointestinal hematoma may be difficult. When indications of obstruction or perforation are considered in an emergency scenario, plain abdominal radiography may be useful. This may indicate normal bowel blockage patterns or symptoms of bowel perforation [2,3]. Colonoscopy by an expert person in uncomplicated instances may be beneficial but not definitive, revealing blue spherical and/or erythematous forms in the submucosal layer with a submucosal mass blocking the luminal space [6]. In our case, during the colonoscopy procedure, the site of the intramural hematoma was identified in the mid-portion of the ascending colon without active bleeding or obvious pathology, and the colonoscope successfully passed through the region.

The gold-standard radiologic method for diagnosis is a contrast-enhanced CT scan [9]. Although a CT scan supports accurate diagnosis in the vast majority of instances, differential diagnosis with other non-cancerous or cancerous gastrointestinal malignancies can be problematic, particularly in occluded or perforated cases [6]. The CT scan should be conducted for both oral and intravenous contrast medium administration, since contrast-enhanced CT alone may disguise the presence of intramural bleeding. Colonic hematoma CT scan diagnostic criteria include eccentric or circumferential wall thickening, intramural hyper-density, lumen constriction, the "coiled spring" sign, the "pseudokidney" sign, and intestinal obstruction [2,3]. In our case, CT imaging characteristics include wall thickening and obstruction without intraperitoneal or retroperitoneal fluid diffusion, which diagnosed the hematoma and colonic obstruction. The additional radiologic method is a barium enema. The signs of intramural hematoma in this radiologic method include "coiled spring," "picket fence signs," or "stacked coin signs" [2].

The recommended treatment for this condition is a conservative approach, as performed in our case [3]. Another option are surgical treatments such as laparoscopy and diagnostic open surgery with resection or bypass of the bowel-involved segment, which are not routinely indicated. The best therapy should be tailored to the various etiologies of intramural hematoma [6]. Non-hemodynamically stable, widespread peritonitis, severe compression or obstruction, and contrast extravasation on a CT scan, a sign of ongoing bleeding, are all indications of urgent laparotomy [2].

Our case has three significant clinical implications. First, a colonic intramural hematoma can develop in individuals who have no predisposing factors, despite it being rarely reported in the literature. The second implication is that our patient was diagnosed with colonic intramural hematoma using two different diagnostic modalities (colonoscopy and CT scan). The third consequence is that our patient was effectively treated with a conservative approach.

## Conclusions

Colonic idiopathic intramural hematoma is an uncommon clinical condition with various triggering causes and non-specific symptoms. A significant level of clinical suspicion is necessary, and early detection helps to reduce complications and unnecessary surgical intervention. Attempts should be made to treat it conservatively. Furthermore, endoscopic treatment may provide a less intrusive option using endoscopic clips or dual knives.
